# Epibiotic Diatoms Are Universally Present on All Sea Turtle Species

**DOI:** 10.1371/journal.pone.0157011

**Published:** 2016-06-03

**Authors:** Nathan J. Robinson, Roksana Majewska, Eric A. Lazo-Wasem, Ronel Nel, Frank V. Paladino, Lourdes Rojas, John D. Zardus, Theodora Pinou

**Affiliations:** 1 The Leatherback Trust, Goldring-Gund Marine Biology Station, Playa Grande, Guanacaste, Costa Rica; 2 Department of Biology, Indiana University-Purdue University Fort Wayne, Fort Wayne, Indiana, United States of America; 3 BioNEM Laboratory, Department of Experimental and Clinical Medicine, University “Magna Graecia” of Catanzaro, Catanzaro, Italy; 4 Division of Invertebrate Zoology, Peabody Museum of Natural History, Yale University, New Haven, Connecticut, United States of America; 5 Institute For Coastal and Marine Research, Nelson Mandela Metropolitan University, Port Elizabeth, South Africa; 6 Department of Biology, The Citadel, 171 Moultrie Street, Charleston, South Carolina, 29407, United States of America; 7 Department of Biological and Environmental Sciences, Western Connecticut State University, Danbury, Connecticut, United States of America; University of Connecticut, UNITED STATES

## Abstract

The macro-epibiotic communities of sea turtles have been subject to growing interest in recent years, yet their micro-epibiotic counterparts are almost entirely unknown. Here, we provide the first evidence that diatoms are epibionts for all seven extant species of sea turtle. Using Scanning Electron Microscopy, we inspected superficial carapace or skin samples from a single representative of each turtle species. We distinguished 18 diatom taxa from these seven individuals, with each sea turtle species hosting at least two diatom taxa. We recommend that future research is undertaken to confirm whether diatom communities vary between sea turtle species and whether these diatom taxa are facultative or obligate commensals.

## Introduction

Sea turtles often harbour complex communities of epibionts [1, 2, 3]. These epibiont communities can provide valuable insights into the hosts’ behaviour [4, 5] and health [6, 7]; however, most studies on sea turtle epibiosis have focused exclusively on macro-epibiota. To date, little is known about the prevalence, and potential ecological function, of sea turtles’ micro-epibiota.

Diatoms are often some of the earliest colonizers on any marine substrate [8] and it has been suggested that sea turtles should harbour epibiotic diatom communities [9]. Moreover, numerous other studies have reported large clumps of periphytic algae growing on the carapace of several sea turtle species [2, 10]. Nevertheless, direct evidence of epibiotic diatoms on sea turtles has only recently been provided on loggerhead turtles *Caretta caretta* [11] and olive ridley *Lepidochelys olivacea* turtles [12]. Consequently, we predict that epibiotic diatoms are likely present on each of the world’s seven extant sea turtle species.

In this study, we used a Scanning Electron Microscopy (SEM) to examine the carapace scutes or skin of flatback *Natator depressus*, green *Chelonia mydas*, hawksbill *Eretmochelys imbricata*, Kemp’s ridley *Lepidochelys kempii*, leatherback *Dermochelys coriacea*, loggerhead *Caretta caretta*, and olive ridley *Lepidochelys olivacea* turtles in search of epibiotic diatoms. Knowledge of the prevalence, characteristic, and diversity of epibiotic diatoms of sea turtles could provide the impetus for more detailed studies into the micro-epibiota of sea turtles.

## Materials and Methods

### Sample Collection

Carapace scutes were opportunistically collected from a single flatback, green, hawksbill, Kemp’s ridley, loggerhead, and olive ridley turtle. Samples were collected from deceased animals that had been stored in either museum or research collections. As leatherback turtles do not have an external shell like the hard-shelled Cheloniidae, we did not collect carapace samples from leatherback turtles. Instead, we collected skin samples from the flippers of nesting turtles using at 6 mm biopsy punches. Full details on sample collection and storage see Table 1.

**Table 1:** Collection and storage details of sea turtle carapace and skin samples.

Species	Scute or Skin	Sampling Location	Collection date and circumstance
Green *Chelonia mydas*	Carapace scute	Hawaii, USA	Collected from a dead-stranded turtle some time prior to 2003. Stored dry at room temperature.^1^
Hawksbill *Eretmochelys imbricata*	Carapace scute	Hawaii, USA	Material confiscated by U.S. Customs perhaps a decade prior to 2002. Stored dry at room temperature. ^1^
Flatback *Natator depressus*	Carapace scute	Northern Territory, Australia	Collected from a dead-stranded turtle in 1981. Stored dry at room temperature.^2^
Kemp’s ridley *Lepidochelys kempii*	Carapace scute	Texas, USA	Collected from a recently dead-stranded turtle in 2003. Stored frozen for an indeterminate period of time before being stored dry and at room temperature. ^1^
Leatherback *Dermochelys coriacea*	Flipper skin	iSimangaliso Wetland Park, South Africa	Collected from a live nesting leatherback turtle in 2013. Stored in 95% non-denatured ethanol at room temperature.^3^
Loggerhead *Caretta caretta*	Carapace scute	Florida, USA	Collected from a dead stranded turtle some time prior to 2009. Stored dry at room temperature. ^1^
Olive ridley *Lepidochelys olivacea*	Carapace scute	Hawaii, USA	Collected from a dead animal that had been caught on a long-line in 2003. Stored dry at room temperature. ^1^

^1^Sample loaned to the Yale Peabody Museum of Natural History by John D. Zardus.

^2^Sample loaned to the Yale Peabody Museum of Natural History by The Bishop Museum. Specimen #8294.

^3^Sample loaned to the Yale Peabody Museum of Natural History by Nathan J. Robinson.

Prior to imaging, the leatherback skin samples were dehydrated in a graded series of hexamethyldisilazane (HMDS) of increasing concentrations until 100% of the latter. HMDS drying for SEM is generally preferred to critical point drying as it is cheaper and it less likely to distort the shape of any microbes of interest [13]. The carapace samples were stored dry and did not need further drying for SEM.

All samples were mounted on aluminium specimen mounts and sputter-coated with carbon. SEM images were collected using a FEI XL-30 field emission gun environmental scanning electron microscope at an accelerating voltage of 10kV, and a Zeiss EM900 transmission electron microscope at 80kV with an objective aperture of 90 μm diameter. Each sample was inspected haphazardly at various magnifications to search for micro-epibionts. We attempted to identify each unique diatom to the lowest taxonomic level by consulting appropriate literature [14, 15, 16, 17, 18, 19, 20, 21, 22,23, 24, 25]. When it was not clear that two diatoms were different taxa, they were considered as one so as not to over-estimate the number of species recorded. As each sample had been stored for various lengths of times and under varying conditions, we only attempted to determine whether micro-epibionts were present or absent—we did not attempt to quantitatively assess the abundance of micro-epibionts. Samples were not cleaned or sonicated prior to imaging as it is expected that these processes would remove the micro-epibionts of interest. SEM work was conducted at the Department of Geology and Geophysics, Yale University.

## Results and Discussion

**Fig 1:**
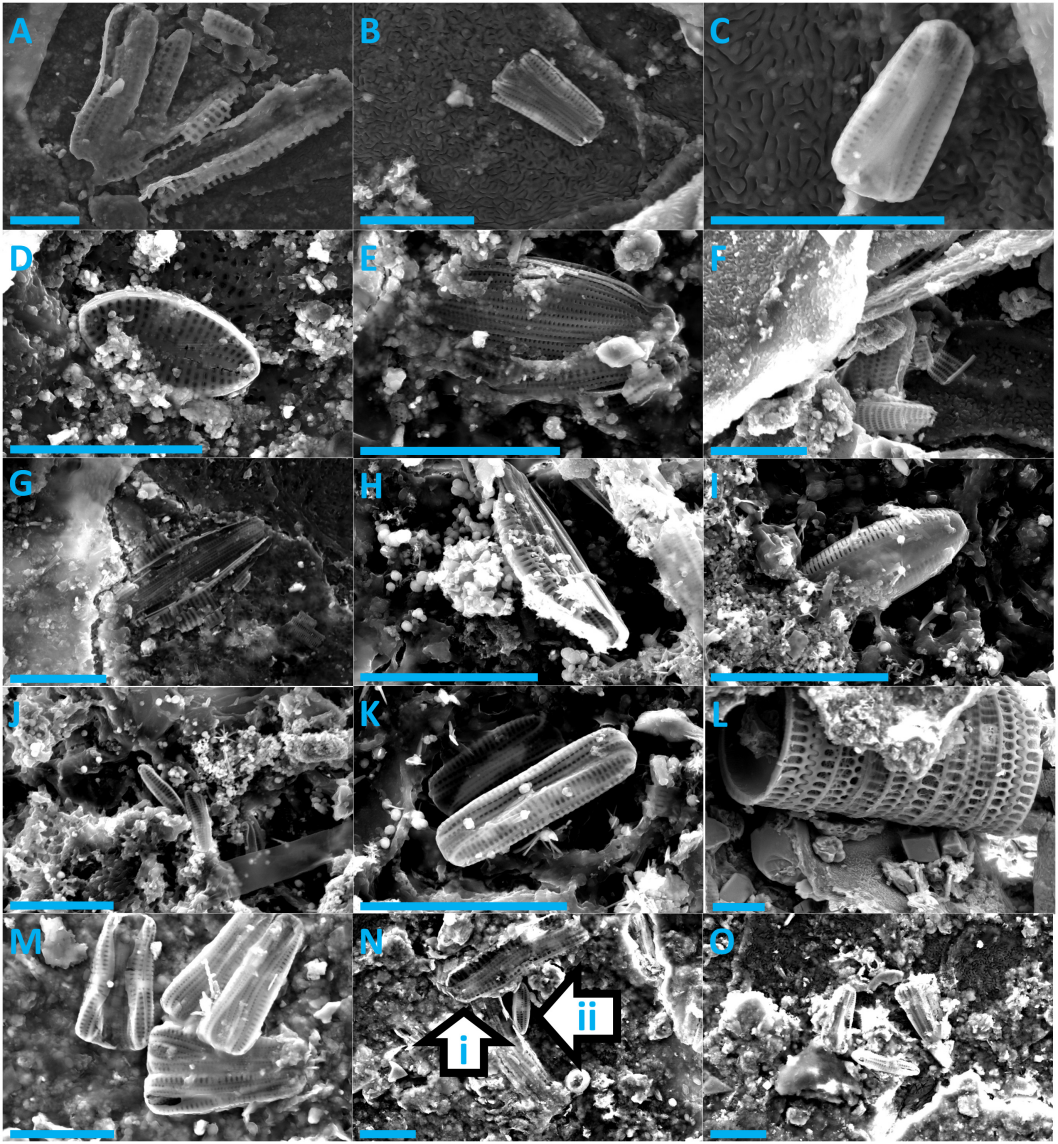
Scanning Electron Microscope images of epibiotic diatoms found on flatback, green, hawksbill, and Kemp’s ridley sea turtles. Flatback turtle: A = *Achnanthes* sp., B & C = *Poulinea* sp. 1; Green turtle: D = *Cocconeis* sp., E = *Amphora* sp. 1, F = Broken pieces of *Amphora* sp. and *Navicula* sp., G = broken pieces of *Amphora* sp.; Hawksbill turtle: H = *Amphora* sp. 2, I = *Amphora* sp. 3, J & K = *Poulinea* sp. 2; Kemp’s ridley turtle: L = *Melosira sol*, M = *Poulinea* sp. 1, N = (i) *Achnanthes* sp. & (ii) *Poulinea* sp. 1, O = *Poulinea* sp. 1. All scale bars are 10 μm.

**Fig 2:**
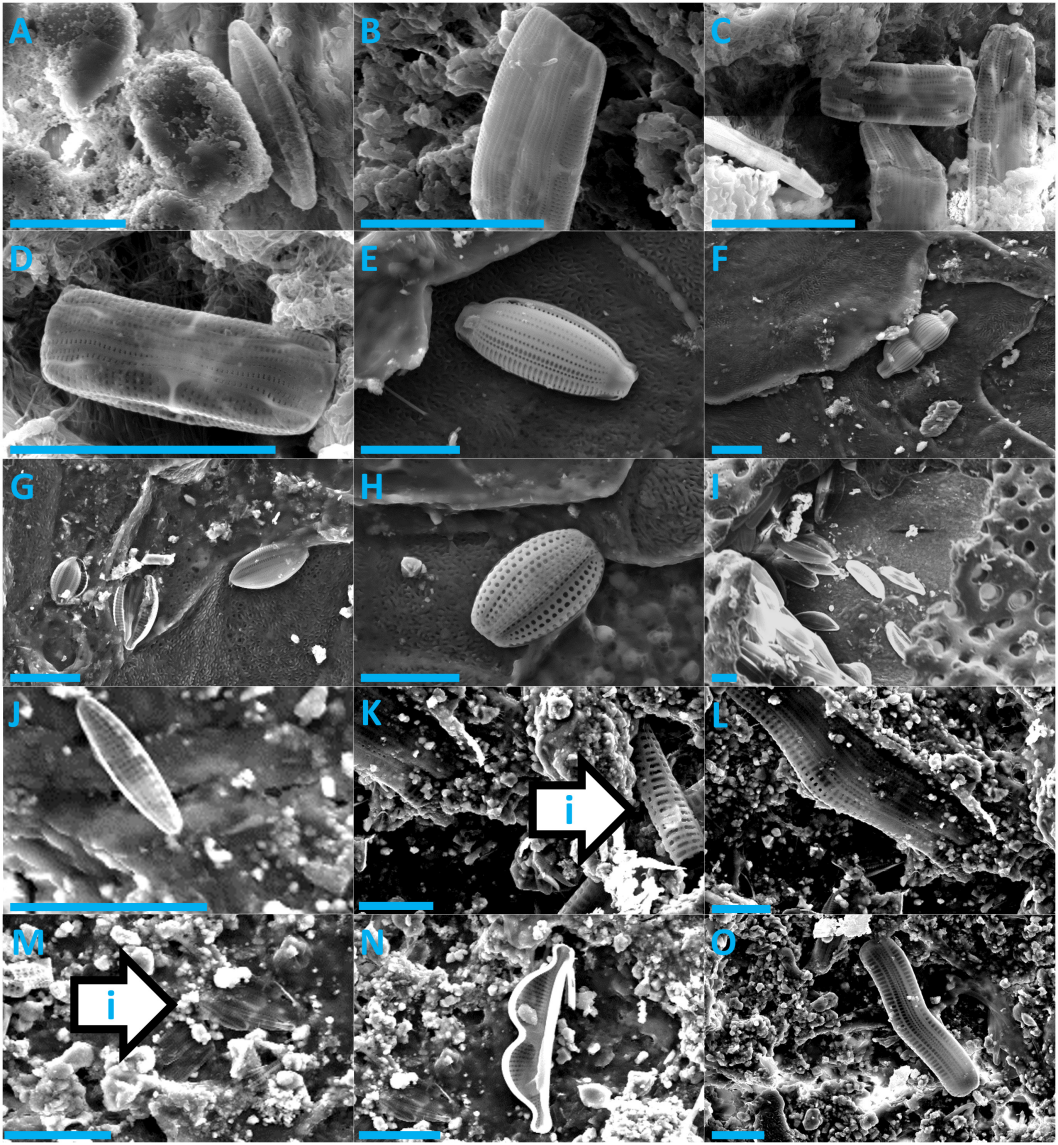
Scanning Electron Microscope images of epibiotic diatoms found on leatherback, loggerhead, and olive ridley sea turtles. Leatherback turtle: A = *Navicula* sp., B, C, and D = *Tursiocola* sp.; Loggerhead turtle: E: *Amphora* sp. 4, F = *Amphora* sp. 5, G = *Amphora* sp. 6, H = *Amphora* sp. 7, I = *Diploneis* sp. and other adnate unknown diatom; Olive ridley turtle: J = Nitzschia sp., K = broken pieces of *Achnanthes* sp (upper left) and possibly other diatom species (arrow), L = *Achnanthes* sp., M = *Poulinea* sp. 3, N = *Amphora* sp. 5, O = *Achnanthes* sp. All scale bars are 10 μm.

Diatoms were present on every sea turtle species (Figs 1 & 2) and from sea turtles from distinct ocean basins (Atlantic, Pacific, and Indian Ocean). We were able to divide all observed diatoms into 18 unique taxa (Table 2). We were only able to identify a single diatom taxon to the species level (*Melosira sol*), all others were only identified to genus level. All diatoms were pennate, with the exception of *Melosira sol*. Adnate forms (*Amphora* spp., *Cocconeis* sp., *Diploneis* sp.) constituted 56% of all identified taxa and erect (*Achnanthes* sp., *Poulinea* spp.) and motile diatoms (*Navicula* sp., *Nitzschia* sp.) constituted 22% and 11%, respectively. The growth form of *Tursiocola* should be considered as uncertain. According to [20, 26], *Tursiocola* spp. has been observed in cetaceans with one end embedded in the epidermis. However, recent observations of live diatoms collected from manatee skin suggest that some *Tursiocola* spp. are highly motile (TA Frankovich, personal communication).

**Table 2:** Diatom taxa found on the seven different sea turtle species.

Species	Diatom species
Green *Chelonia mydas*	*Cocconeis* sp., *Amphora* sp. 1, and possibly fragments from a *Navicula* sp.
Hawksbill *Eretmochelys imbricata*	*Amphora* sp. 2, *Amphora* sp. 3, and *Poulinea* sp. 2
Flatback *Natator depressus*	*Achnanthes* sp., and *Poulinea* sp. 1
Kemp’s ridley *Lepidochelys kempii*	*Melosira sol*, *Poulinea* sp. 1, and *Achnanthes* sp.
Leatherback *Dermochelys coriacea*	*Navicula* sp., and *Tursiocola* sp.
Loggerhead *Caretta caretta*	*Amphora* sp. 4, *Amphora* sp. 5, *Amphora* sp. 6, *Amphora* sp. 7, *Diploneis* sp., and an unknown adnate species
Olive ridley *Lepidochelys olivacea*	*Nitzchia* sp., *Achnanthes* sp., *Poulinea* sp. 3, and *Amphora* sp. 5

When compared to descriptions of known diatom taxa, many of the 18 diatom taxa seen in this study differed in important aspects of their morphology. For example, the diatom shown in Fig 2I could not be satisfactorily assigned to any existing genus. So far, only one *Poulinea* sp. has been described [25]) and, due to differences in the central area, shape, and number of areolae, we believe that the taxa in this study to do not belong to this species. A detailed taxonomic analysis of sea turtle diatoms would therefore be a productive avenue for future research.

Many diatom taxa were only observed on a single host; however, three diatom species were found on multiple host species. *Achnanthes* sp. was found on flatback, Kemp’s ridley, and olive ridley turtles. *Amphora* sp. 5 was found on loggerhead and olive ridley turtles, and *Poulinea* sp. 1 was found on flatback and Kemp’s ridley turtles. Even with the limited sample size used in this study, the presence of comparable diatom taxa on different host species from different localities suggests that that diatom assemblages on sea turtles may be very similar in structure and composition regardless of the hosts’ species or geographic location.

Epibiosis in the marine environment is primarily facultative in nature [8] and this is probably the case with the majority of diatoms documented here. As such the survival of these epibiotic diatoms may not depend upon settling on a sea turtle host and they may be present in the micro-plankton or on a variety of substrates. Indeed, taxa such as *Melirosa sol* and *Nitzchia* sp. are often living in the plankton and may have been present in the surrounding water at the time that the sea turtle tissue sample was collected. Nevertheless, there is also evidence that some diatom taxa observed in our study are obligate sea turtle epibionts. The diatom genera, *Poulinea* and *Chelonicola*, have only recently been described from olive ridley carapaces and have not been observed elsewhere [25]. *Tursiocola* spp. may also be an obligate epibiont, even though it is not exclusive to sea turtles and has been observed on cetaceans [21], freshwater and marine turtles [11, 27 respectively] and the West Indian manatee *Trichechus manatus* [24].

**Fig 3:**
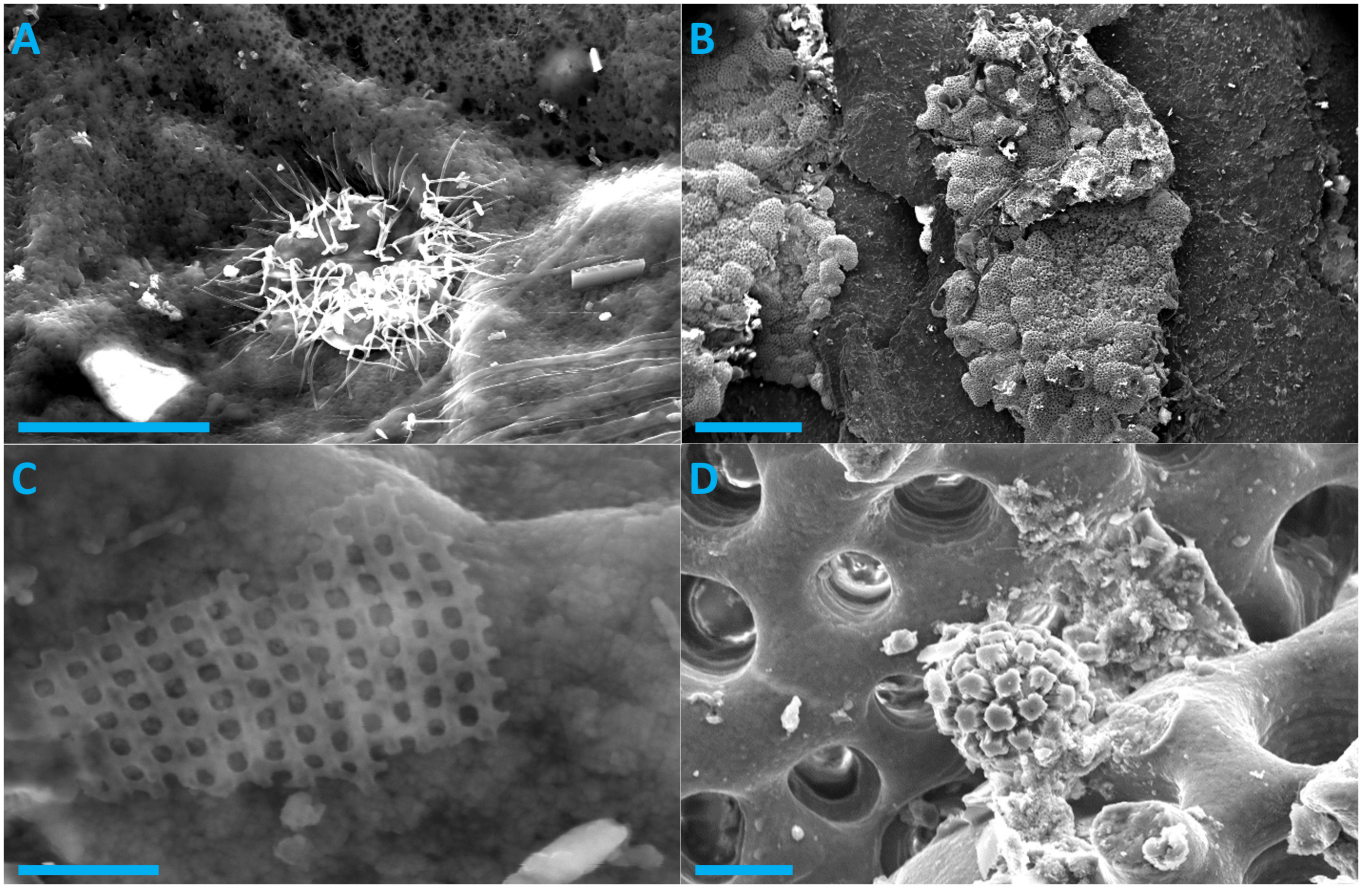
Scanning Electron Microscope images of other epibiotic organisms found on loggerhead turtles (A and B). C and D are epibionts found on the formanifera in image B. Scale bars are 10, 500, 1, and 10 μm in images A, B, C, and D respectively.

In addition to epibiotic diatoms, other micro-organisms were also discovered by SEM. Although we could not unequivocally identify these organisms we presume them to be a hystrichosphere from a dinoflagellate (Fig 3A) and an encrusting foraminifera (Fig 3B). Furthermore, evidence of a diatom valve (Fig 3C) and a coccolithophore (Fig 3D) were also found on the foraminifera in Fig 3B. Interestingly, these micro-organisms were all found on the carapace sample from a loggerhead turtle. Loggerhead turtles generally host the most diverse macro-epibiont communities [1] and thus it stands to reason that loggerhead turtles also host the most diverse micro-epibiont communities.
